# P2Y12 Inhibitor or Aspirin Monotherapy for Chronic Coronary Disease: A Nationwide Cohort Study

**DOI:** 10.1155/cdr/2715470

**Published:** 2025-12-16

**Authors:** Minyoul Baik, Jimin Jeon, Joonsang Yoo, Jinkwon Kim

**Affiliations:** ^1^ Department of Neurology, Yongin Severance Hospital, Yonsei University College of Medicine, Yongin-si, Gyeonggi-do, Republic of Korea, yonsei.ac.kr

**Keywords:** aspirin, chronic coronary disease, p2y12 inhibitor, percutaneous coronary intervention

## Abstract

**Background:**

The 2024 European Society of Cardiology (ESC) guideline newly recommended clopidogrel as a safe and effective alternative to aspirin monotherapy in patients with chronic coronary disease (CAD). We aimed to validate the 2024 ESC guideline recommendation by comparing the prognosis of patients with chronic CAD treated with P2Y12 inhibitor monotherapy and aspirin monotherapy.

**Methods:**

This retrospective cohort study included patients with chronic CAD (18 months after percutaneous coronary intervention [PCI] with drug‐eluting stents [DES] in 2019–2023), based on a nationwide health claims database in Korea. A 1:1 propensity score matching was performed between P2Y12 inhibitors and aspirin monotherapy groups. The primary composite outcome included all‐cause death, myocardial infarction, ischemic stroke, and major bleeding. Stratified Cox regression models were used to compare risks between groups.

**Results:**

Of 127,127 patients with chronic CAD (mean age: 63.1 years; 73.7% men), 84,440 (66.4%) patients received P2Y12 inhibitor monotherapy, and 42,727 (33.6%) received aspirin monotherapy. After propensity score matching, 42,692 pairs were generated. During a median follow‐up of 3 years, P2Y12 inhibitor monotherapy did not reduce the risk of the primary composite outcome (hazard ratio [HR]: 0.98; 95% confidence interval [CI]: 0.92–1.05; *p* = 0.577) compared with aspirin monotherapy. In secondary analyses, P2Y12 inhibitors showed a trend toward reduced major bleeding (HR: 0.86; 95% CI: 0.72–1.02; *p* = 0.082) and a significant reduction in major gastrointestinal bleeding (HR: 0.79; 95% CI: 0.63–0.97; *p* = 0.027).

**Conclusions:**

Among Korean patients with chronic CAD in the long‐term maintenance period after PCI using DES, P2Y12 inhibitor monotherapy demonstrated overall outcomes comparable with aspirin monotherapy, with a potential advantage in reducing bleeding, particularly of gastrointestinal origin. These findings support the safety and feasibility of P2Y12 inhibitor monotherapy, in line with the 2024 ESC guideline recommendations, while emphasizing the need for further prospective studies to confirm its clinical benefit.

## 1. Introduction

Chronic coronary artery disease (CAD) is a heterogeneous condition that includes both obstructive and nonobstructive forms, with or without previous myocardial infarction (MI) or revascularization, as well as ischemic heart disease diagnosed through noninvasive examinations, and chronic angina syndromes [1, 2]. CAD is a chronic, most often progressive disease, and is therefore serious, even during clinically silent periods [1, 2]. Given that CAD shows dynamic process, modifying the progression of chronic CAD through lifestyle adjustments, pharmacological therapies, and invasive interventions, is important [1, 2].

Long‐term maintenance of antiplatelet monotherapy is the standard of care for patients with chronic CAD [1–3]. Aspirin monotherapy has traditionally been the drug of choice, with a Class 1 recommendation (level of evidence A) in both 2023 American College of Cardiology/American Heart Association (ACC/AHA) and 2019 European Society of Cardiology (ESC) guidelines for chronic CAD [1, 2, 4]. Recent studies have shown that a short course (1–3 months) of dual antiplatelet therapy (DAPT) followed by P2Y12 inhibitor monotherapy after percutaneous coronary intervention (PCI) have proven to be effective [5–7], highlighting the role of P2Y12 inhibitors in the management of CAD. More importantly, a randomized trial and meta‐analyses suggested that P2Y12 inhibitor monotherapy may offer superior efficacy with acceptable safety compared with aspirin in patients with chronic CAD [8–12]. Based on these results, the latest updated 2024 ESC guideline for chronic CAD recommended clopidogrel as a safe and effective alternative to aspirin monotherapy, with a Class 1 recommendation (level of evidence A) [3].

In this study, we aimed to validate the aforementioned 2024 ESC guideline recommendation [3], by investigating the comparative effectiveness and safety profiles of P2Y12 inhibitor monotherapy versus aspirin in Korean patients with chronic CAD, 18 months after PCI with a drug‐eluting stent (DES), using a nationwide population‐based database.

## 2. Materials and methods

### 2.1. Ethics Statement

This study was approved by the Institutional Review Board of Yongin Severance Hospital, Yonsei University Health System (No. 9‐2023‐0231). The requirement for informed consent was waived because of the retrospective nature of the study, which was based on an anonymous health insurance claims database. The research reported in this study adhered to the Strengthening the Reporting of Observational Studies in Epidemiology (STROBE) Guidelines.

### 2.2. Data Source and Participants

This retrospective cohort study used data obtained from the Health Insurance Review and Assessment Service (HIRA) of Korea. The Korean National Health Insurance Service (NHIS) is a government‐run, mandatory, single‐payer insurance system that provides comprehensive medical coverage for approximately 97% of the Korean population. The remaining 3%, representing the lowest‐income group, is covered by the Medical Aid program. Both systems are integrated into a single claims database managed by the HIRA, which oversees all claims and quality assessments in Korea [13, 14]. As a result, the HIRA database effectively represents the entire Korean population. From the HIRA database, we screened patients who underwent PCI with DES between January 2015 and January 2021. The inclusion criterion was admission with insurance claims for PCI (M6551‐4, M6561‐7, and M6571‐2) and DES (J5083 and J8083). Patients with chronic CAD were operationally defined as patients who remained free of clinical events during the 18 months after PCI [8]. Given the nature of claims data, minor ischemic or bleeding events may not be captured reliably, and a substantial proportion of patients were still prescribed DAPT at 18 months despite no documented clinical events. Therefore, rather than relying on the timing of DAPT to SAPT transition, we adopted a conservative definition based on a sufficiently long event‐free period. The 18‐month duration was inspired by the prior randomized trial and chosen as one of the most conservative time points to identify a relatively stable population of patients with chronic CAD for comparison of antiplatelet strategies [8]. To investigate the effect of antiplatelet monotherapy in chronic CAD, the index date was defined as 18 months after the date of PCI admission (Supplementary Figure S1); therefore, patients who were followed up for less than the index date were excluded. The database for the current study has been available since 2014; thus, there was at least a 1‐year washout period before PCI admission for all patients.

Patients with prior PCI or coronary artery bypass graft surgery before the index PCI, those who were admitted for > 30 days during baseline PCI admission, those who were not prescribed DAPT after baseline PCI admission, and those younger than 18 years were excluded. To investigate patients with chronic CAD, we excluded those who experienced a primary outcome, death, or loss to follow‐up before the index date (18 months after baseline PCI admission). Among patients with chronic CAD, we further excluded those who were prescribed DAPT, other antiplatelet agents, or oral anticoagulants, as well as those without a prescription of either aspirin or P2Y12 inhibitors on the index date. DAPT was defined as the combination of aspirin and a P2Y12 inhibitor, including clopidogrel, prasugrel, and ticagrelor. Because the use of antiplatelet agents was defined as taking the medication for at least 21 days within 30 days after the index date patients who were censored within 30 days after the index date were inevitably excluded to ensure equal time for all patients to claim prescriptions for medications and to minimize the risk of immortal time bias.

### 2.3. Independent Variable and Covariates

Comorbidities and medications were identified using ICD‐10 diagnosis codes and Anatomical Therapeutic Chemical Classification System codes (Supplementary Table S1). Using prescription records in the HIRA database, the medications of the study participants, including antiplatelet agents, statins, antihypertensive agents (beta‐blockers, renin–angiotensin–aldosterone system [RAAS] inhibitors, calcium channel blockers [CCBs], and spironolactone), and PPIs were identified and classified.

P2Y12 inhibitor or aspirin monotherapy in chronic CAD, the independent variable in this study, was defined as the use of antiplatelet agents on the index date (18 months after baseline PCI admission). The use of antiplatelet agents was defined as the use of medication for at least 21 days within 30 days after the index date (Supplementary Figure S1). Clopidogrel is classified as a classic P2Y12 inhibitor, whereas prasugrel and ticagrelor are classified as potent P2Y12 inhibitors.

Finally, patients were categorized according to the use of a P2Y12 inhibitor or aspirin as monotherapy, which was the independent variable in this study. The use of other concomitant medications was also determined by taking the medication for at least 21 days within 30 days after the index date. The timeline of this study and detailed information based on the claims data are presented in Supplementary Figure S1 and Supplemental Table S1.

### 2.4. Outcomes and Follow‐Ups

To evaluate the effect of antiplatelet monotherapy during the maintenance period of chronic CAD, patients were followed up from the index date, corresponding to a follow‐up period of 18 months after baseline PCI admission (Supplementary Figure S1). Primary composite outcomes included all‐cause death, MI, ischemic stroke, and major bleeding. MI was defined as admission with a related primary diagnostic code and accordant claims for treatment (Supplemental Table S1). Ischemic stroke was defined as an admission with a related primary diagnostic code and brain imaging claims. Major bleeding was a composite of major gastrointestinal (GI) bleeding and hemorrhagic stroke. Major GI bleeding was defined as an admission with a related primary diagnostic code and claims for red blood cell transfusion claims. Hemorrhagic stroke was defined as admission with a related primary diagnostic code and brain imaging claims. Any GI bleeding was defined using the same diagnostic codes as major GI bleeding without the need for transfusion. Detailed definitions based on claims data are provided in Supplemental Table S1.

After the index date, the patients were followed up until either the development of the primary outcome, loss of NHIS eligibility due to emigration, death, or the end of the study period (July 31, 2023), whichever came first.

### 2.5. Statistical analyses

To reduce the potential confounding effects of different baseline characteristics between the P2Y12 inhibitor and aspirin groups, we conducted a 1:1 propensity score matching (PSM). Propensity scores were estimated using a logistic regression model based on the use of P2Y12 inhibitors or aspirin, which included age, sex, insurance type, hypertension, diabetes, heart failure, prior MI, prior stroke, peripheral artery disease, chronic kidney disease, liver disease, baseline PCI indication, number of stents, DAPT at baseline PCI admission, DAPT duration, and concomitant medications including statins, beta‐blockers, RAAS inhibitors, CCBs, spironolactone, and PPIs. For the matching, a nearest‐neighbor‐matching algorithm was applied based on a difference of 0.1 times the standard deviation of the logit‐transformed propensity scores. These covariates were primarily selected based on previous landmark randomized trials [8, 15] and were further refined considering the characteristics and limitations specific to the Korean claims database. Covariate balance was evaluated using standardized mean differences, with a standardized difference of < 0.1 considered adequate balance. The validity of the proportional hazards assumption was tested using Schoenfeld residuals. Within the matched cohort, cumulative incidence curve for the primary composite outcome was plotted according to monotherapy (P2Y12 inhibitor vs. aspirin), and a stratified log‐rank test was performed. To evaluate the effect of the P2Y12 inhibitor compared with aspirin on the risk of the primary composite outcome, we applied a stratified Cox regression model to the PSM cohort and calculated the hazard ratio (HR) and 95% confidence interval (CI). To evaluate the differential effect of the classic P2Y12 inhibitor and potent P2Y12 inhibitors compared with aspirin, we performed Cox regression analyses with the following group comparisons: aspirin vs. clopidogrel, and aspirin vs. potent P2Y12 inhibitors (ticagrelor or prasugrel). For secondary outcome analysis, we constructed individual cause‐specific stratified Cox regression models for each outcome. In the secondary outcome analysis, if a competing event occurred before the outcome of interest, the observation was censored at the time of the competing event. Statistical analyses were performed using SAS (Version 9.4.2; SAS Institute) and R (Version 3.5.1; R Foundation for Statistical Computing). Statistical significance was set at *p* < 0.05.

## 3. Results

### 3.1. Study Population and Baseline Characteristics

**Figure 1:**
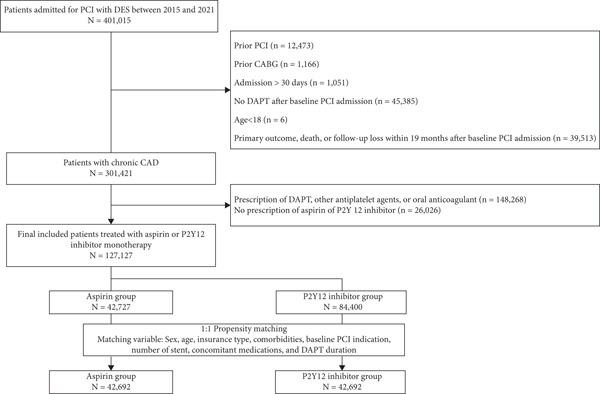
Flow diagram of patient inclusion. CABG, coronary artery bypass graft surgery; CAD, coronary artery disease; DAPT, dual antiplatelet therapy; DES, drug‐eluting stent; PCI, percutaneous coronary intervention.

Between 2015 and 2021, 401,015 patients with PCI using DES were identified (Figure 1). After applying the exclusion criteria, 127,127 patients with chronic CAD were finally included (mean age ± standard deviation: 63.1 ± 11.2 years; and 62,956 [73.7%] were men). Of these, 84,400 (66.4%) received P2Y12 inhibitor monotherapy and 42,727 (33.6%) received aspirin monotherapy during the chronic stage after PCI (Figure 1). Compared with the aspirin monotherapy group, patients receiving P2Y12 inhibitor monotherapy were older, less often male, and had a higher prevalence of cardiovascular comorbidities, including hypertension, diabetes mellitus, heart failure, prior MI, prior stroke, and chronic kidney disease; however, PCI for acute MI was more common in the aspirin monotherapy group (all *p* < 0.05; Supplement Table S2).

After a 1:1 PSM between aspirin and P2Y12 inhibitor monotherapy, 42,692 patients were selected from each group of the matched cohort (Table 1). The matched cohort was well balanced in terms of the absolute standardized mean difference < 0.1 (Table 1).

**Table 1:** Baseline characteristics of patients according to monotherapy after propensity score matching.

**Variables**	**Aspirin** **(** **N** = 42,692**)**	**P2Y12 inhibitor** **(** **N** = 42,692**)**	**SMD**
Sex, male	31429 (73.62)	31527 (73.85)	0.005
Age, years	63.08 ± 11.20	63.03 ± 11.20	0.004
Insurance type			0.002
Health insurance	40905 (95.81)	40918 (95.84)	
Medical aid	1787 (4.19)	1774 (4.16)	
Comorbidities			
Hypertension	33909 (79.43)	33897 (79.40)	0.001
Diabetes mellitus	13398 (31.38)	13279 (31.10)	0.006
Heart failure	12492 (29.26)	12294 (28.80)	0.010
Prior MI	3113 (7.29)	3119 (7.31)	0.001
Prior stroke	4872 (11.41)	4752 (11.13)	0.009
PAOD	1824 (4.27)	1821 (4.27)	< 0.001
Chronic kidney disease	3225 (7.55)	3204 (7.50)	0.002
Liver disease	1789 (4.19)	1773 (4.15)	0.002
Baseline PCI indication			0.009
Acute MI	17408 (40.78)	17215 (40.32)	
Others	25284 (59.22)	25477 (59.68)	
Number of stents, median [IQR]	1 [1‐1]	1 [1‐1]	0.002
DAPT at PCI admission			0.006
Clopidogrel	27327 (64.01)	27452 (64.30)	
Potent P2Y12 inhibitor	15365 (35.99)	15240 (35.70)	
DAPT duration ≥ 12months	22096 (51.76)	22154 (51.89)	0.003
Concomitant medications			
Statins	41078 (96.22)	41079 (96.22)	< 0.001
Beta blockers	24692 (57.84)	24688 (57.83)	< 0.001
RAAS inhibitors	26047 (61.01)	25981 (60.86)	0.003
CCBs	14918 (34.94)	14892 (34.88)	0.001
Spironolactone	1694 (3.97)	1662 (3.89)	0.004
PPIs	10273 (24.06)	10320 (24.17)	0.003

*Note:* Data is presented as numbers (%) or means ± standard deviations. All absolute standardized mean difference values were less than 0.1 in the propensity score‐matched cohort.

Abbreviations: CCBs, calcium channel blockers; DAPT, dual antiplatelet therapy; MI, myocardial infarction; PAD, peripheral artery disease; PCI, percutaneous coronary intervention; PPI, proton pump inhibitor; RAAS, renin–angiotensin–aldosterone system; SMD, standardized mean difference.

### 3.2. Primary and Secondary Outcomes

During long‐term follow‐up (median: 3.02 years, IQR: 1.48–4.78), the primary composite outcome occurred in 2953 patients (6.9%) receiving P2Y12 inhibitor monotherapy and 3629 patients (8.5%) receiving aspirin monotherapy (HR: 0.98, 95% CI: 0.92–1.05, *p* = 0.577, Figure 2a and Table 2). The proportional hazards assumption for the two groups was assessed by computing the Schoenfeld residuals and was not violated (*p* = 0.329).

Figure 2Cumulative incidence of clinical outcomes. There was no significant difference in (a) the primary composite outcome (*p* = 0.577), or (b) MI (*p* = 0.362) between groups. P2Y12 inhibitor monotherapy showed a trend toward a lower risk of (c) major bleeding (*p* = 0.082) and a significantly lower risk of (d) major GI bleeding (*p* = 0.027). Time 0 indicated the index date, which was 18 months after the baseline PCI admission. GI, gastrointestinal; MI, myocardial infarction; PCI, percutaneous coronary intervention.

**Figure:**
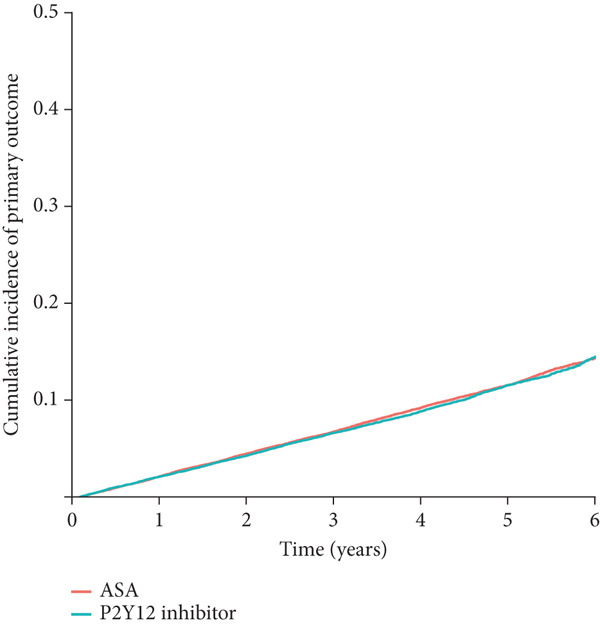
(a)

**Figure:**
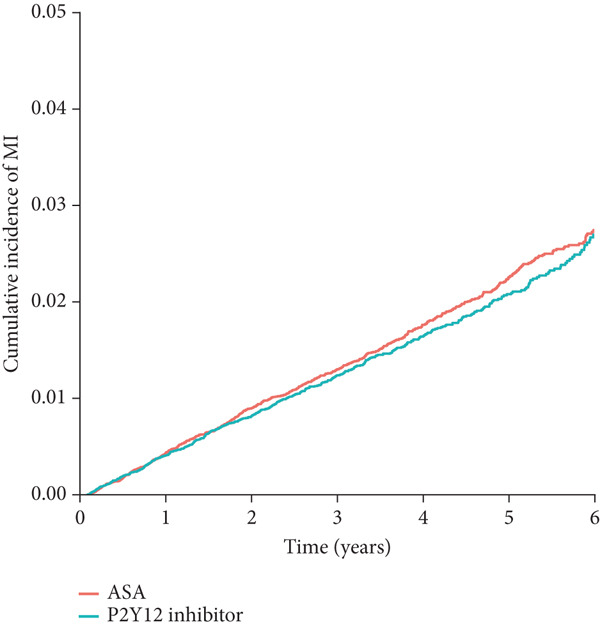
(b)

**Figure:**
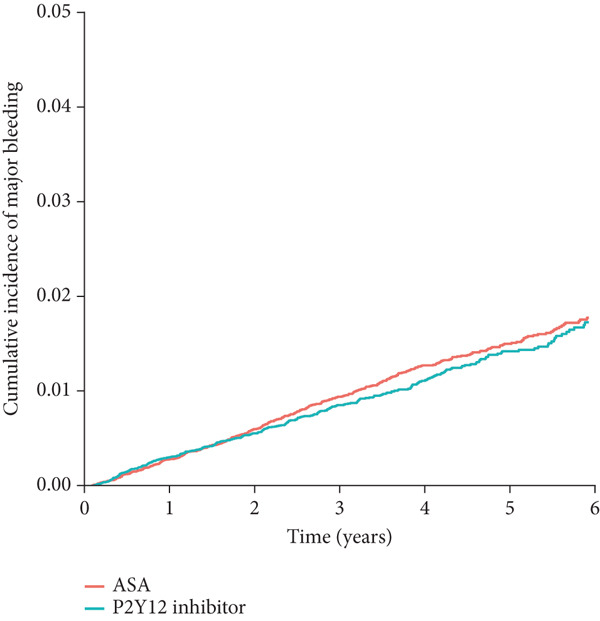
(c)

**Figure:**
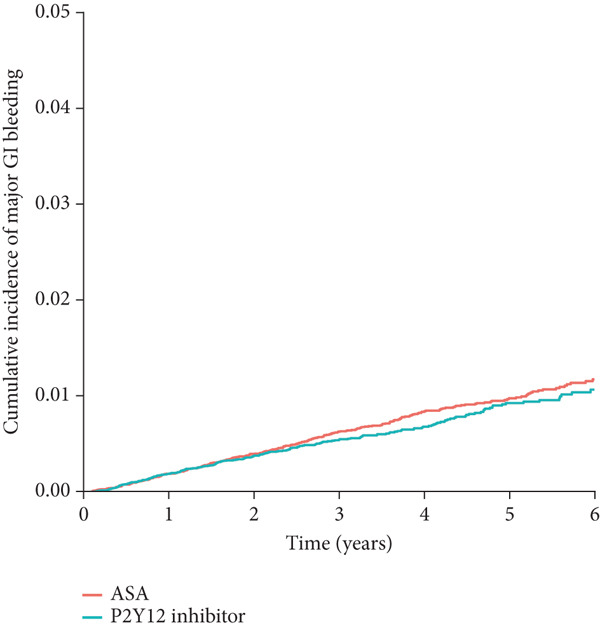
(d)

**Table 2:** Risk of clinical outcomes by aspirin and P2Y12 monotherapy.

	**Events/total**		**HR (95% CI)**	**p** **value**
	**P2Y12 inhibitor**	**Aspirin**		
Primary outcome^a^	2953/42692	3629/42692	0.98 [0.92–1.05]	0.577
Secondary outcomes				
All‐cause death	1601/42692	1937/42692	1.04 [0.95–1.13]	0.389
MI	527/42692	660/42692	0.94 [0.81–1.08]	0.362
Stroke	605/42692	739/42692	0.98 [0.85–1.13]	0.777
Ischemic	476/42692	586/42692	0.97 [0.83–1.13]	0.691
Hemorrhagic	129/42692	153/42692	1.02 [0.75–1.39]	0.875
Major bleeding	349/42692	446/42692	0.86 [0.72–1.02]	0.082
Major GI bleeding	220/42692	293/42692	0.79 [0.63–0.97]	0.027
Any GI bleeding	635/42692	717/42692	1.01 [0.90–1.15]	0.826

Abbreviations: CI, confidence interval; GI, gastrointestinal; HR, hazard ratio; MI, myocardial infarction.

^a^Composite of all‐cause death, MI, ischemic stroke, and major bleeding. A 1:1 propensity score matching was performed and stratified Cox regression analysis was performed in the matched cohort.

Regarding the type of P2Y12 inhibitors used (clopidogrel and potent P2Y12 inhibitors), patients receiving potent P2Y12 inhibitors had better outcomes compared with those receiving aspirin (*p* = 0.004); however, there was no significant difference between the clopidogrel and aspirin groups (*p* = 0.830) (Supplemental Table S3).

In the secondary outcome analyses, there was no significant difference in the risk of MI between the groups (HR: 0.94; 95% CI: 0.85–1.13; *p* = 0.362; Figure 2b and Table 2). P2Y12 inhibitor monotherapy showed a trend toward a lower risk of major bleeding (HR: 0.86; 95% CI: 0.72–1.02; *p* = 0.082; Figure 2c and Table 2) and was associated with a significantly lower risk of major GI bleeding (HR: 0.79; 95% CI: 0.63–0.97; *p* = 0.027; Figure 2d and Table 2).

## 4. Discussion

Using a nationwide claims database in Korea, we investigated the effects of P2Y12 inhibitor monotherapy versus aspirin in patients with chronic CAD, 18 months after PCI with DES. P2Y12 inhibitor monotherapy showed comparable overall outcomes to aspirin for the primary composite endpoint. It was associated with a trend toward lower rates of major bleeding and a significantly reduced risk of major GI bleeding. These findings support the safety and feasibility of P2Y12 inhibitor monotherapy in the long‐term maintenance stage after PCI with DES, consistent with the updated 2024 ESC guideline recommendation [3].

Aspirin has long been the cornerstone of antiplatelet therapy in chronic CAD, as recommended by the 2023 ACC/AHA and 2019 ESC guidelines [1, 2, 4]. However, most supporting evidence is derived from older trials [4]. Until recently, the HOST‐EXtended Antiplatelet Monotherapy (HOST‐EXAM) study was the only randomized trial to directly investigate this issue [8]. In Korean patients 6–18 months after PCI with DES, clopidogrel monotherapy significantly reduced the 24‐month composite of all‐cause death, nonfatal MI, stroke, readmission due to acute coronary syndrome, and severe bleeding compared with aspirin [8], and this benefit persisted over 5 years [25]. Recent patient‐level meta‐analyses also showed that P2Y12 inhibitor monotherapy was superior to aspirin for long‐term prevention in patients with established CAD, particularly after PCI [9, 12]. Reflecting these evidences, the recently updated 2024 ESC guideline recommends clopidogrel as a safe and effective alternative to aspirin monotherapy for chronic CAD [3]. Moreover, while our study was ongoing, Smart Angioplasty Research Team: Choice of Optimal Anti‐Thrombotic Strategy in Patients Undergoing Implantation of Coronary Drug‐Eluting Stents (SMART‐CHOICE) 3 trial showed that among high‐risk Korean patients after PCI, clopidogrel significantly lowered the composite risk of all‐cause death, MI, and stroke compared with aspirin [15].

In contrast, our nationwide claims‐based study, which included a homogenous cohort of Korean patients with chronic CAD defined as 18 months after PCI using DES, found that P2Y12 inhibitor monotherapy (over 95% clopidogrel) yielded composite outcomes comparable with aspirin monotherapy. The discrepancy from randomized trials favoring clopidogrel may reflect differences between trial settings and real‐world practice [16, 17], as well as residual confounding inherent to our retrospective design. Population ancestry alone is unlikely to explain this divergence, given that both HOST‐EXAM and SMART‐CHOICE 3 trials were also conducted in Korean populations. Furthermore, inclusion of potent P2Y12 inhibitors does not account for the difference, as our supplementary analyses suggested that potent P2Y12 inhibitors were associated with better outcomes than aspirin, whereas clopidogrel was not. Previous studies have demonstrated the superiority of potent P2Y12 inhibitors over clopidogrel in DAPT among patients with acute coronary syndrome [18, 19], and in strategies involving early transition to monotherapy after PCI [6, 20]. Although our findings should be interpreted with caution, the observed trend toward better outcomes with potent P2Y12 inhibitors compared with aspirin may provide preliminary evidence supporting their potential role as monotherapy in chronic CAD after PCI with DES. These findings underscore the need for future randomized trials specifically evaluating potent P2Y12‐inhibitor monotherapy in chronic CAD.

Our study showed that P2Y12 inhibitor monotherapy had a similar risk of MI, but a potential advantage in reducing bleeding, particularly of GI origin compared with aspirin monotherapy. The role of aspirin has recently been questioned mainly because of its higher bleeding risk, especially of GI origin, and the emergence of newer antiplatelets such as P2Y12 inhibitors [21]. In a substudy of the GLOBAL LEADERS randomized trial, which compared 23 months ticagrelor monotherapy following 1 month of DAPT with 12 months of aspirin monotherapy following 12 months of DAPT [6], a time‐dependent treatment effect was observed [22]: ticagrelor monotherapy reduced MI and stent thrombosis but increased minor bleeding compared with aspirin after 1 year post‐PCI [22]. A post hoc analysis landmark analysis during 12–24 months after PCI similarly found fewer ischemic events, mainly MI, with ticagrelor but a numerically higher rate of serious bleeding [23]. The HOST‐EXAM trial demonstrated that clopidogrel reduced acute coronary syndrome, stroke, major, or any GI bleeding compared with aspirin [8]. The SMART‐CHOICE 3 trial showed the benefits of clopidogrel over aspirin in reducing MI, and upper GI clinical event; however, no difference in major bleeding or GI bleeding [15]. Meta‐analyses in patients with established atherosclerosis including CAD, ischemic stroke, or peripheral artery disease, have indicated that P2Y12 inhibitor monotherapy lowers ischemic risk, particularly MI, without increasing severe bleeding [10, 11]. Patient level meta‐analyses of randomized trials have shown that, compared with aspirin, P2Y12 inhibitor monotherapy reduced MI risk without raising major bleeding among PCI‐treated patients (DES > 95%) [12]. Similarly, in established CAD, P2Y12 inhibitor monotherapy reduced MI, hemorrhagic stroke, and any GI bleeding [9]. Focusing on patients who underwent PCI with DES, a network meta‐analysis demonstrated that P2Y12 inhibitor monotherapy following DAPT significantly reduced MI with comparable major bleeding risk relative to aspirin monotherapy [24]. In summary, most studies suggest that P2Y12 inhibitor monotherapy lowers MI risk; however, findings for stroke, bleeding, and mortality remain inconsistent [8, 9, 11, 15, 25, 22–24]. These heterogeneous results, together with our findings, underscore the need for further research to clarify the benefits of P2Y12 inhibitor monotherapy.

This study had several limitations. First, because the data used health claims, not originally made for research purposes, information regarding which vessel was stented and the territory of MI could not be evaluated; therefore, we could not assess the risk of repeat revascularization or stent thrombosis. Although PSM was performed using multiple covariates, the absence of these important unmeasured variables, together with the retrospective design, may limit the interpretation and generalizability of our findings. Furthermore, because of the inherent limitations of claims‐based data, outcomes such as bleeding related to surgical procedures or trauma could not be adequately assessed. Second, a relatively small number of patients (less than 3%) treated with potent P2Y12 inhibitors were included. Therefore, we could not conclusively compare the effects of clopidogrel and potent P2Y12 inhibitors, such as ticagrelor or prasugrel. The differential effectiveness and safety between clopidogrel and potent P2Y12 inhibitors as monotherapy in chronic CAD remain important unresolved questions that warrant further investigation. Third, this study was conducted using the Korean claims database, and therefore, the generalizability of our findings to other ethnicities may be limited. Notably, to date, only two head‐to‐head randomized trials—HOST‐EXAM and SMART‐CHOICE 3—have compared clopidogrel monotherapy (a P2Y12 inhibitor) with aspirin monotherapy in patients with chronic CAD after PCI, and both trials were conducted in Korea [8, 15]. Recent patient‐level meta‐analysis and recent guidelines do not include head‐to‐head randomized trial data addressing this issue in non‐Asian populations [1, 3, 12]. It is also noteworthy that in our claims data, P2Y12 inhibitor monotherapy was prescribed nearly twice as often as aspirin monotherapy (84,400 vs. 42,727), suggesting a clear preference among Korean physicians, even before the publication of updated guidelines [1, 3]. Although the underlying reasons for this preference remain uncertain, it may have contributed to the initiation and design of the aforementioned randomized trials [8, 15]. Future studies involving more ethnically diverse populations are warranted to confirm the generalizability of these findings. Despite these limitations, this study had several strengths. First, we included homogenous patients with definite chronic CAD, defined as 18 months after PCI using DES [8]. Second, we included a substantial number of patients with chronic CAD (*N* = 127,127) in the real‐world data, which exceeds the sample size of the recent patient‐level meta‐analysis (*N* = 16,117) [12]. Third, we minimized the risk of selection bias with its nationwide scope. Finally, we confirmed the potential clinical benefits of P2Y12 inhibitors using PSM.

In conclusion, among Korean patients with chronic CAD, defined as 18 months after PCI with DES, P2Y12 inhibitor monotherapy demonstrated comparable overall outcomes with aspirin monotherapy, with a trend toward reduced bleeding, particularly of GI origin. These findings align with the recently updated 2024 ESC guideline recommendation.

## Conflicts of Interest

M.B. received a research grant from Daewoong and HK inno. N Pharmaceuticals. J.K. received a research grant from Myung In Pharmaceutical. J.Y. and J.J. declare no conflicts of interest.

## Author Contributions

M.B., J.Y., and J.K. contributed to the concept and design of the study; M.B., J.J., and J.K., contributed to the acquisition and analysis of the data; and M.B., J.Y., and J.K. contributed to drafting the manuscript and preparing the figures.

## Funding

This study was supported by a faculty research grant from the Yonsei University College of Medicine (6‐2024‐0144), and the National Research Foundation of Korea grant funded by the Korean Government (MSIT) (Grant No. RS‐2024‐00345524).

## Supporting information


**Supporting Information** Additional supporting information can be found online in the Supporting Information section. Supplemental Figure S1. Schematic timelines of the study. Supplemental Table S1. Definition of variables based on health claim data. Supplemental Table S2. Baseline characteristics of patients according to monotherapy before propensity score matching. Supplemental Table S3. Risk for primary outcome according to P212 inhibitor type compared to aspirin.

## Data Availability

The dataset utilized in this study was restricted and provided by HIRA. As data access was strictly limited to the scope of this research, the dataset is not available for public access. Researchers, however, can submit requests to the Korean Health Insurance Review and Health Big Data Hub (https://opendata.hira.or.kr).
