# Supplementary material for: P2Y12 Inhibitor or Aspirin Monotherapy for Chronic Coronary Disease: A Nationwide Cohort Study
Source: Cardiovasc Ther. 2025 Dec 16;2025:2715470. doi: 10.1155/cdr/2715470 (PMC12721736; doi:10.1155/cdr/2715470)
Supplement: Supplementary file 1 — Supporting Information Additional supporting information can be found online in the Supporting Information section. Supplemental Figure S1. Schematic timelines of the study. Supplemental Table S1. Definition of variables based on health claim data. Supplemental Table S2. Baseline characteristics of patients according to monotherapy before propensity score matching. Supplemental Table S3. Risk for primary outcome according to P212 inhibitor type compared to aspirin. [file CDR-2025-2715470-s001.docx]

Supplemental Material

**P2Y12 inhibitor or aspirin monotherapy for chronic coronary disease: a nationwide cohort study**

Minyoul Baik, MD^1^; Jimin Jeon, MS^1^; Joonsang Yoo, MD, PhD ^1^; Jinkwon Kim, MD, PhD^1^

^1^Department of Neurology, Yongin Severance Hospital, Yonsei University College of Medicine, Yongin-si, Gyeonggi-do, South Korea

**Supplemental Figure S1.** Schematic timelines of the study


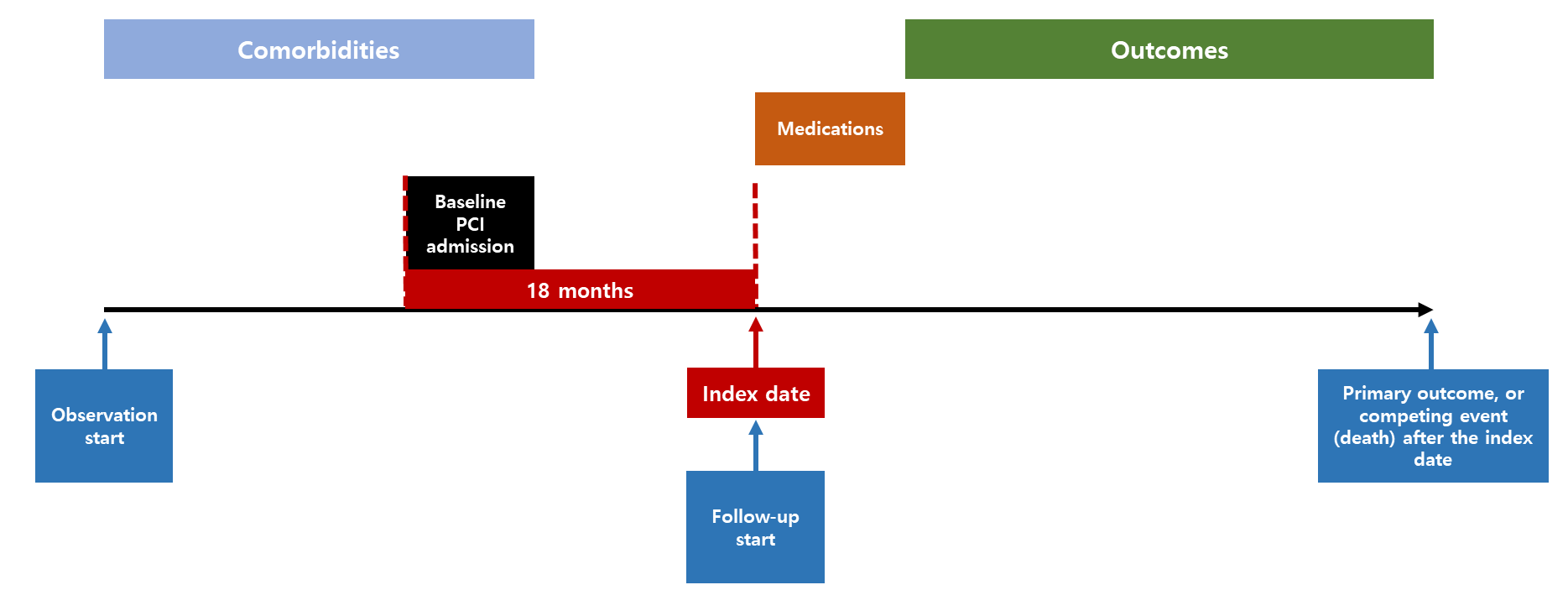


Antiplatelet monotherapy and concomitant medications were defined as taking the medication for at least 21 days within 30 days after the index date.

PCI, percutaneous coronary intervention.

**Supplemental Table S1.** Definition of variables based on health claim data

|  | **ICD-10 and claim codes** |
| --- | --- |
| **Inclusion/exclusion criteria** |  |
| PCI with DES | Claims for PCI (M6551–4, M6561–4, M6571–2) and claim for DES (J5083, J8083) |
| **Comorbidities** |  |
| Hypertension | I10–15; and prescription of antihypertensive drug |
| Diabetes mellitus | E11-E14; and prescription of antidiabetic drugs |
| Heart failure | I11.0, I13.0, I13.2, and I50 |
| Prior MI | I21-23, I25.2 |
| Prior stroke | I60-64, I69 |
| PAOD | I70.2, I73.8, I73.9, I74.3, I74.4, I74.5, I74.8, and I74.9 |
| Chronic kidney disease | N18–19 |
| Liver disease | C22, K70.2, K70.3, K70.4, K74.6, K70.1, B18.0-2 at least 2 times |
| Index PCI indication |  |
| Acute MI | Diagnosis of I21–22, or claims for PCI for acute MI (M6553, M6565) during index PCI admission |
| Number of stents | Total number of claimed stents during index PCI admission (DES [J5083, J5084, J8083], BMS [J5231, J8231, J5232]) |
| **Outcome** |  |
| Primary composite outcome | All-cause death, MI, ischemic stroke, and major bleeding |
| MI | Admission with the primary diagnosis of I21–22 and claims for PCI (M6551–4, M6561–7, and M6571–2), CABG (O1640, O1641, O1642, O1645, O1646, O1647, O1648, O1649, OA640, OA641, OA642, OA647, OA648, OA649), or thrombolytics (alteplase, tenecteplase, urokinase) |
| Ischemic stroke | Admission with the primary diagnosis of I63 and brain imaging (CT or MRI)≥1 |
| Major bleeding | Major GI bleeding or Hemorrhagic stroke |
| Severe GI bleeding | Admission with the primary diagnosis of K22.6, K25.0, K25.2, K25.4, K25.6, K26.0, K26.2, K26.4, K26.6, K27.0, K27.2, K27.4, K27.6, K28.0, K28.2, K28.4, K28.6, K29.0, K62.5, K66.1, K92.0, K92.1, K92.2, I85.0, I98.3, K62.5, K22.11, K31.81 and claims of red blood cell transfusion (X2021, X2022, X2031, X2032, X2131, X2132, X2091, X2092, X2111, X2112, X2515, X2512) |
| Hemorrhagic stroke | Admission with the primary diagnosis of I60-62 and brain imaging (CT or MRI)≥1 |
| Any GI bleeding | Admission with the primary diagnosis of K22.6, K25.0, K25.2, K25.4, K25.6, K26.0, K26.2, K26.4, K26.6, K27.0, K27.2, K27.4, K27.6, K28.0, K28.2, K28.4, K28.6, K29.0, K62.5, K66.1, K92.0, K92.1, K92.2, I85.0, I98.3, K62.5, K22.11, K31.81 |

ATC, Anatomical Therapeutic Chemical Classification System; CABG, coronary artery bypass graft surgery; DES, drug-eluting stent; ICD, International Classification of Diseases; GI, gastrointestinal; UGI, upper GI; MI, myocardial infarction; PCI, percutaneous coronary intervention.

**Supplemental Table S2.** Baseline characteristics of patients according to monotherapy before propensity score matching

| **Variables** | **Aspirin**  **(N=42727)** | **P2Y12 inhibitor**  **(N=84400)** | **P value** | **SMD** |
| --- | --- | --- | --- | --- |
| Sex, male | 31461 (73.63) | 59697 (70.73) | <0.001 | 0.065 |
| Age, years | 63.07±11.21 | 64.27±11.19 | <0.001 | 0.107 |
| Insurance type |  |  | <0.001 | 0.034 |
| Health insurance | 40940 (95.82) | 80276 (95.11) |  |  |
| Medical aid | 1787 (4.18) | 4124 (4.89) |  |  |
| Comorbidities |  |  |  |  |
| Hypertension | 33929 (79.41) | 68340 (80.97) | <0.001 | 0.039 |
| Diabetes mellitus | 13409 (31.38) | 27742 (32.87) | <0.001 | 0.032 |
| Heart failure | 12497 (29.25) | 26314 (31.18) | <0.001 | 0.042 |
| Prior MI | 3113 (7.29) | 6820 (8.08) | <0.001 | 0.030 |
| Prior stroke | 4873 (11.40) | 11759 (13.93) | <0.001 | 0.076 |
| PAOD | 1824 (4.27) | 4266 (5.05) | <0.001 | 0.037 |
| Chronic kidney disease | 3226 (7.55) | 7059 (8.36) | <0.001 | 0.030 |
| Liver disease | 1789 (4.19) | 3629 (4.30) | <0.001 | 0.006 |
| Baseline PCI indication |  |  | <0.001 | 0.071 |
| Acute MI | 17432 (40.80) | 31510 (37.33) |  |  |
| Others | 25295 (59.20) | 52890 (62.67) |  |  |
| Number of stents, median [IQR] | 1 [1-1] | 1 [1-1] | 0.185 | 0.008 |
| DAPT at PCI admission |  |  | <0.001 | 0.193 |
| Clopidogrel | 27327 (63.96) | 61504 (72.87) |  |  |
| Potent P2Y12 inhibitor | 15400 (36.04) | 22896 (27.13) |  |  |
| Concomitant medications |  |  |  |  |
| Statins | 41078 (96.14) | 82062 (97.23) | <0.001 | 0.061 |
| Beta blockers | 24717 (57.85) | 46349 (54.92) | <0.001 | 0.059 |
| RAAS inhibitors | 26063 (61.00) | 52226 (61.88) | 0.002 | 0.018 |
| CCBs | 14925 (34.93) | 30971 (36.70) | <0.001 | 0.037 |
| Spironolactone | 1694 (3.96) | 3948 (4.68) | <0.001 | 0.035 |
| PPIs | 10273 (24.04) | 26007 (30.81) | <0.001 | 0.152 |
| DAPT duration |  |  | 0.001 | 0.020 |
| <12month | 20622 (48.26) | 39910 (47.29) |  |  |
| ≥12month | 22105 (51.74) | 44490 (52.71) |  |  |

Data is presented as numbers (%) or means ± standard deviations.

CCBs, calcium channel blockers; DAPT, dual antiplatelet therapy; MI, myocardial infarction; PAD, peripheral artery disease; PCI, percutaneous coronary intervention; PPI, proton pump inhibitor; RAAS, renin-angiotensin-aldosterone system; SMD, standardized mean difference.

**Supplemental Table S3.** Risk for primary outcome according to P2Y12 inhibitors type compared to aspirin

|  | **Events/total** | **HR [95% CI]** | **P–value** |
| --- | --- | --- | --- |
| **Aspirin** | 3629/42692 | ref | - |
| **Clopidogrel** | 2844/40675 | 0.99 [0.95–1.04] | 0.830 |
| **Potent P2Y12 inhibitors** | 109/2017 | 0.75 [0.62–0.91] | 0.004 |

Data were obtained using a Cox proportional hazard regression model for outcome development.

CI, confidence interval; HR, hazard ratio.
